# Assessing the Food Environment of a Rural Community: Baseline Findings From the Heart of New Ulm Project, Minnesota, 2010–2011

**DOI:** 10.5888/pcd11.130291

**Published:** 2014-03-06

**Authors:** Raquel F. Pereira, Abbey C. Sidebottom, Jackie L. Boucher, Rebecca Lindberg, Rebecca Werner

**Affiliations:** Author Affiliations: Abbey C. Sidebottom, Allina Health, Minneapolis, Minnesota; Jackie L. Boucher, Rebecca Lindberg, Minneapolis Heart Institute Foundation, Minneapolis, Minnesota; Rebecca Werner, New Ulm Medical Center, New Ulm, Minnesota.

## Abstract

**Introduction:**

Changes in the food environment in the United States during the past few decades have contributed to increased rates of obesity, diabetes, and heart disease. Improving the food environment may be an effective primary prevention strategy to address these rising disease rates. The purpose of this study was to assess the consumer food environment of a rural community with high rates of obesity and low levels of fruit and vegetable consumption. Findings were used to identify food environment intervention strategies to be implemented as part of a larger community-based heart disease prevention program.

**Methods:**

We used the Nutrition Environment Measures Survey for Restaurants (NEMS-R) and Stores (NEMS-S) to assess 34 restaurants, 3 grocery stores, and 5 convenience stores in New Ulm, Minnesota.

**Results:**

At least half of the restaurants offered nonfried vegetables and 100% fruit juice. Only 32% had at least 1 entrée or 1 main dish salad that met standards for “healthy.” Fewer than half (41%) had fruit available and under one-third offered reduced-size portions (29%) or whole-grain bread (26%). Grocery stores had more healthful items available, but findings were mixed on whether these items were made available at a lower price than less healthful items. Convenience stores were less likely to have fruits and vegetables and less likely to carry more healthful products (except milk) than grocery stores.

**Conclusion:**

Baseline findings indicated opportunities to improve availability, quality, and price of foods to support more healthful eating. A community-wide food environment assessment can be used to strategically plan targeted interventions.

## Introduction

Changes in the food environment in the United States during the past few decades have contributed to the obesity epidemic and associated rising rates of diabetes and heart disease. These changes include an increase in the number of food establishments (1) and an increase in the availability of processed and convenience foods (2). Simultaneously, there has been an increase in the frequency of eating out (3) and in the percentage of food dollars spent on meals away from home (1,4). Increased eating out is associated with eating foods that are higher in fat, saturated fat, and sodium, and lower in fiber than foods prepared at home (4). Portion sizes have increased in chain restaurants, fast-food outlets, and stores (5,6).

Modifying the food environment may be among the most effective strategies to promote more healthful food choices among US residents and reduce the prevalence of chronic diseases (2,7,8). Addressing the food environment in rural areas may be of particular importance. Residents of rural areas have a higher prevalence of diabetes, obesity, coronary heart disease, and poverty than residents of urban areas (9,10). Furthermore, rural food environments may be characterized by less access to supermarkets, which generally sell more healthful foods than convenience stores (11,12). This lack of access to healthful foods prevents healthful eating and may result in weight gain (13).

The purpose of this study was to conduct a comprehensive assessment of the consumer food environment in a rural community, with a focus on where adults frequently make food decisions. The findings of the assessment will be used to guide intervention strategies for a community-based heart disease prevention program.

## Methods

### Context

This study took place in the context of a population-based research and demonstration project. Hearts Beat Back: The Heart of New Ulm Project (HONU) is a 10-year initiative designed to deliver interventions at individual, institutional, and community levels that reduce the rate of myocardial infarctions and cardiovascular disease risk factors identified in the INTERHEART Study (14) among residents aged 40 to 79 years of New Ulm, Minnesota (15,16). The community is located in a predominantly agricultural region approximately 100 miles southwest of the Minneapolis–St. Paul metropolitan area. HONU is a collaborative partnership of Allina Health, the Minneapolis Heart Institute Foundation, and the community of New Ulm. Results from baseline heart health screenings identified high obesity rates, high prevalence of metabolic syndrome, and low fruit and vegetable consumption as priority risk factors to address (16). Because HONU targets adults, food environment assessment activities conducted at baseline focused on consumer environments where adults frequently make food decisions.

### Screening instruments

The Nutrition Environment Measures Survey for Restaurants (NEMS-R) (17) was used to assess restaurants; grocery and convenience stores were assessed using the Nutrition Environment Measures Survey for Stores (NEMS-S) (18). When this assessment was being planned (2009–2010), these instruments were the most widely disseminated and validated assessment tools available to measure consumer nutrition environments (19). Both tools examine their respective environments for “healthy” meals or availability of “healthier” options given the common types of foods offered and consumed. Determination of “healthy” was based on federal guidelines when the tool was developed (2005) (29).

The NEMS-R was created to assess dietary factors in the restaurant food environment related to risk of major chronic diseases such as obesity, diabetes, and cardiovascular disease (17). The instrument assesses the “relative healthfulness” of foods and beverages available on main and children’s menus and factors that may support or challenge more healthful eating (17). The tool consists of a menu review, a restaurant observational visit, and interview with restaurant staff as needed. The instrument assesses the availability of entrées, main dish salads, side dishes (ie, fruit without added sugar, nonfried vegetables without sauce or toppings, baked chips, whole grain bread), and beverages (ie, diet soda, 100% fruit juice, and 1% or nonfat milk) that meet the criteria for being designated as “healthy.” Criteria for designating menu items as “healthy” came from government recommendations for a healthful diet at the time of development and were primarily based on calories, fat, saturated fat. For example “healthy” entrées were defined as having 800 or fewer calories, 30% or less calories from fat, and 10% or less calories from saturated fat, or as having a regulated healthy designation (eg, low fat, light). If insufficient information was available to determine whether the item met these guidelines, the item was assumed to be unhealthy. In addition, the instrument includes measures of barriers to and facilitators of healthful eating and measures of pricing and signs related to promotion of healthier or less healthy foods (17). Reliability testing found high interrater and test-retest reliability (κ > 0.80) and strong construct validity (17).

Measures included in the NEMS-S were selected on the basis of the types of food products that contribute the most fat and calories to the American diet, and those most recommended for healthful eating as defined by federal guidelines and health professional organizations at the time of tool development (29). The instrument includes 11 indicators of food categories (healthier options in parentheses): fruit (fresh), vegetables (fresh), milk (skim/low-fat), ground beef (lean), hot dogs (lean), frozen dinners (reduced-calorie), baked goods (low-fat), beverages (soda: diet/low-calorie/juice: 100% juice), bread (whole grain), chips (baked), and cereal (higher fiber). Measures for each component include availability of healthier options, with more points if additional varieties of the healthier option are available; quality of produce (acceptable/unacceptable based on overripeness/bruising); and price. Price is collected as the absolute price per pound or per item for fruit and vegetables. For the other 9 items, the price measures are comparisons between the cost of the healthier options and the regular options, such as low-fat compared with whole milk and lean versus regular ground beef. A lower price for a healthier item is scored positively and a higher price for a healthier item subtracts a point. Composite scores are calculated for availability, quality, and price, and an overall score combines the 3 dimensions. A higher score indicates higher quality, availability, or lower prices for the healthier items (18). The instrument was tested in 88 stores and found high values for interrater and test-retest reliability (18).

### Data collection

The HONU team received training from the NEMS team in 2009. The 2-day training consisted of classroom sessions and practice in the restaurant and store settings (19).

A letter was mailed to all restaurants, convenience stores, and grocery stores in New Ulm explaining the purpose of the assessment and how to opt out. One coffee shop indicated it did not want the assessment done, and the researchers decided not to assess 1 bakery because of its limited menu. This project was determined to be exempt by Allina’s institutional review board.

Data were collected during spring 2010 (convenience stores), fall 2010 (grocery stores), and late 2010 and early 2011 (restaurants); most assessments were conducted by the project’s registered dietitian and a health educator. The 2 raters conducted cross-assessments of a few restaurants to ensure they were completing assessments comparably. NEMS-S data were recorded onto paper copies of the screening tool. Results were entered into an Excel spreadsheet, provided by the NEMS-S developers, which calculated scores. Because the NEMS-R is longer and more complex than the NEMS-S and because there were many more restaurants to assess than stores, a database was developed in Microsoft Access to duplicate the NEMS-R instrument, with structured data entry forms and reports using scoring algorithms. The NEMS-R data were entered into the database via laptops at each restaurant.

### Data analysis

Data analysis for the NEMS-R was done using SPSS version 18.0 (IBM Corporation, Armonk, New York). Frequencies were used to describe restaurant characteristics and results of the NEMS-R. The proportion of healthy item availability on kids’ menus was calculated only for restaurants with a kids’ menu. To compare practices between restaurant types, cross-tabulations were conducted. Because of small sample size, statistical tests were not conducted. For the NEMS-S data, frequencies and average summary scores were calculated in the spreadsheet provided by the NEMS developers for grocery stores and convenience stores separately.

## Results

### Restaurants

Of the 34 restaurants assessed, there were more independent (56%) than chain (44%) restaurants (Table 1). All independent restaurants were sit-down. There was limited variety in the type of cuisines offered, most (71%) being general American, pizza, or burgers.

**Table 1:** Characteristics of Restaurants (n = 34), Heart of New Ulm Project, Minnesota, 2010–2011

Characteristic	No. (%)
**Type of restaurant**	
Sit-down^a^	22 (65)
Fast-food^b^	10 (29)
Other	2 (6)
**Independent or chain**	
Independent	19 (56)
Chain	15 (44)
**Combined restaurant/service type**	
Independent sit-down	19 (56)
Chain sit-down	5 (15)
Chain fast-food	10 (29)
**Cuisine**	
General/mixed American	14 (41)
Pizza	6 (18)
Burgers	4 (12)
Mexican	3 (9)
Asian (mixed)/Chinese	2 (6)
Other	5 (15)

a Full table service or bars with full menu.

b Minimal service and food supplied quickly after ordering.

Healthier practices, as defined by the NEMS-R, most commonly offered (ie, in at least 80% of restaurants) were the availability of diet soda and no extra charge for a shared entrée (Table 2). More than two-thirds of the restaurants offered 1% or nonfat milk. At least half of the restaurants offered nonfried vegetables and 100% fruit juice. Only 32% of restaurants had at least 1 entrée or 1 main dish salad that met the NEMS-R standards of “healthy.” Even fewer (21%) identified the healthy entrées on their menu. Fewer than half (41%) had fruit without added sugar available and less than one-third offered reduced-size portions (29%), whole-grain bread (26%), or a baked chip option (12%).

**Table 2:** NEMS-R Measures, by Restaurant Type, Heart of New Ulm Project, Minnesota, 2010–2011

Measure	Sit Down (n = 24)	Fast Food (n = 10)	Total (n = 34)

	No. (%)		
**Main dishes/entrees**			
Healthy entrée available	5 (21)	6 (60.0)	11 (32)
Number of healthy entrees available			
0	19 (79)	4 (40)	23 (68)
1–2	0	2 (20)	2 (6)
3–5	4 (17)	1 (10)	5 (15)
≥6	1 (4)	3 (30)	4 (12)
**Main-dish salads**			
Healthy main-dish salads available	5 (21)	6 (60)	11 (32)
Number of healthy salads available			
0	19 (79)	4 (40)	23 (68)
1–2	3 (12)	4 (40)	7 (21)
3–5	1 (4)	1 (10)	2 (6)
≥6	1 (4)	1 (10)	2 (6)
**Specific food availability**			
Availability of fruit without added sugar	11 (46)	3 (30)	14 (41)
Nonfried vegetable availability	15 (62)	5 (50)	20 (59)
Baked chip availability	2 (8)	2 (20)	4 (12)
Whole-grain bread availability	7 (29)	2 (20)	9 (26)
**Beverages**			
Diet soda availability	24 (100)	10 (100)	34 (100)
100% Fruit juice availability	15 (62)	6 (60)	21 (62)
1% or Nonfat milk availability	20 (83)	5 (50)	25 (74)
**Kids’ menu**			
Available	9 (38)	7 (70)	16 (47)
Healthy choice available^a^	1 (11)	3 (43)	4 (25)
100% Fruit juice available^a^	6 (67)	4 (57)	10 (62)
1% or Nonfat milk available^a^	7 (78)	4 (57)	11 (69)
**Facilitators of healthful eating**			
Nutrition information on menu	1 (4)	4 (40)	5 (15)
Healthy entrées identified on menu	3 (12)	4 (40)	7 (21)
Reduced-sized portions available^b^	8 (33)	2 (20)	10 (29)
Healthy requests encouraged	2 (8)	3 (30)	5 (15)
Salad bar	8 (33)	1 (10)	9 (26)
**Barriers to healthful eating**			
Large portions encouraged	1 (4)	3 (30)	4 (12)
Menu discourages special requests	1 (4)	0	1 (3)
“All-you-can-eat” or “unlimited” available	6 (25)	1 (10)	7 (21)
**Pricing**			
Combination meal cheaper than sum of price of individual items	0	1 (10)	1 (3)
Healthy entrees less expensive than regular entrees	0	0	0
No charge for shared entrée	21 (88)	9 (90)	30 (88)
Designated smaller portion less expensive than regular portion	0	0	0
**Signage**			
Nutrition information posted	1 (4)	5 (50)	6 (18)
Healthy options highlighted	1 (4)	3 (30)	4 (12)
Healthy eating encouraged	2 (8)	2 (20)	4 (12)
Unhealthy eating encouraged	3 (12)	4 (40)	7 (21)
Overeating encouraged	1 (4)	1 (10)	2 (6)

Abbreviation: NEMS-R, Nutrition Environment Measures Survey for Restaurants.

a Percentages calculated on the basis of restaurants that had a kids’ menu.

b Reduced sizes do not include offerings at restaurants where varying-sized food items, such as pizza, burger sandwiches, or beverages, are considered standard.

Findings are mixed regarding which type of restaurant had healthier practices (Table 2). Fast-food restaurants were more likely than sit-down restaurants to offer healthy entrées and healthy main-dish salads; however, they were also more likely to have nutrition information available, allowing raters to calculate whether items met the criteria for healthy entrées. Such information was generally not available in the nonchain restaurants. Fast-food restaurants were also less likely than sit-down restaurants to offer an “all-you-can-eat” option and had more signs encouraging healthful requests. However, fast-food restaurants were more likely to encourage larger portions than sit-down restaurants. Some healthier practices that were more common among sit-down restaurants include the availability of 1% or nonfat milk, 100% juice, nonfried vegetables, fruit without added sugar, a salad bar, and reduced-size portions.

### Grocery and convenience stores

The NEMS-S assessment was conducted in all 3 grocery stores and all 5 convenience stores (Table 3). Grocery store results indicate availability of all of the healthier options for each of the food indicators and mixed findings on the measure of price comparability. Some items were likely to have a lower price for the healthier item (ie, skim/low-fat milk, higher-fiber cereal, and low-fat baked goods), while other healthier items were generally associated with a higher price (ie, whole-wheat bread, baked chips, lean ground beef, lean hot dogs) or varied by store (ie, low-calorie frozen dinners). The grocery stores, on average, achieved nearly the highest possible scores for availability and quality but lower average price comparability score.

**Table 3:** Availability of More Healthful Options, Pricing Features, and NEMS-S Scores for Grocery and Convenience Stores, Heart of New Ulm Project, Minnesota, 2010–2011

Availability of Food Types	Convenience Stores (n = 5)	Grocery Stores (n = 3)

	No. (%)	
**Any fruit, no. of varieties**	2 (40)	3 (100)
0	3 (60)	0
1–4	1 (20)	0
5–9	1 (20)	1 (33)
10	0	2 (67)
**Any vegetables, no. of varieties**	1 (20)	3 (100)
0	4 (80)	0
1–4	1 (20)	0
5–9	0	0
10	0	3 (100)
**Skim/low-fat milk**	5 (100)	3 (100)
**Lean ground beef**	0	3 (100)
**Low-fat or fat-free hot dogs**	1 (20)	3 (100)
**Reduced-fat frozen dinners**	2 (40)	3 (100)
**Diet soda**	5 (100)	3 (100)
**100% Fruit juice**	5 (100)	3 (100)
**Low-fat baked goods**	1 (20)	3 (100)
**100% Whole-grain bread**	1 (20)	3 (100)
**≥2 Varieties of whole-grain bread**	0	3 (100)
**Baked/low-fat chips**	4 (80)	3 (100)
**Availability of healthier cereal**	3 (60)	3 (100)
**Selected price comparison measures**		
**Milk**		
Lower price for lowest fat	3 (60)	3 (100)
Same price for whole and skim	2 (40)	0
Higher price for low fat	0	0
**Frozen dinners**		
Lower price for reduced fat	0	1 (33)
Higher price for reduced fat	1 (20)	1 (33)
**Baked chips**		
Lower price for baked chips	0	0
Higher price for baked chips	1 (20)	2 (67)
**Cereal**		
Lower price for healthier cereal	0	2 (67)
Higher price for healthier cereal	2 (40)	1
**Bread**		
Lower price for whole wheat	0	0
Higher price for whole wheat	1 (20)	2 (67)
**Baked goods**		
Lower price for low fat	1 (20)	3 (100)
Higher price for low fat	1 (20)	0
**Ground beef**		
Lower price for lean meat	NA	0
Higher price for lean meat	NA	3 (100)
**Composite scores (total possible)**	**Mean (SD)**	
Availability (30)	10.6 (6.4)	28 (1)
Price (18)	2.3 (0.6)	1.7 (1.5)
Quality (6)	2.7 (1.8)	6 (0)
Total (54)	13 (6.4)	35.7 (2.3)

Abbreviations: NEMS-S, Nutrition Environment Measures Survey for Stores; NA, not applicable; SD, standard deviation.

Convenience stores were less likely to have fruits and vegetables and less likely to carry healthier products (except milk) than grocery stores (Table 3). Availability, quality, and total scores were lower for convenience stores than grocery stores. As with grocery stores, the convenience stores scored low on price. At convenience stores, because there were fewer healthier items available, it was harder to compare prices of healthier and regular items.

## Discussion

This study is unique because it assessed nearly all restaurants, convenience, and grocery stores in 1 rural community. Results indicated that practices that are more supportive of healthful eating can be implemented in these 3 food outlet sectors in this rural food environment. Several feasible healthful practices were observed in fewer than half of the restaurants and convenience stores. Restaurants generally lacked several basic practices to encourage more healthful food choices, such as making healthy entrées and main dish salads, as well as fruits, baked chips, and whole grains more readily available. The availability and promotion of healthful side dishes or menu items was also not a common practice. In stores, the opportunity exists to test effectiveness and feasibility of a positive pricing structure for healthier items.

Much of research on the food environment in rural and urban areas focuses on access to or density of different types of restaurants or stores (13,20–22). These studies provide insight into the association of access or types of outlets and obesity but may provide less guidance for planning interventions in restaurants or stores. One study using food permit records to categorize healthfulness of food sources found that most establishments were categorized as “unhealthy” or “undetermined”; authors concluded that future research should establish health values of foods in different types of food establishments (21). Few studies have examined food offerings in restaurants in rural areas. One study conducted nutrition analysis on meals from children’s menus and found that lower-fat, smaller portions were more available in fast-food than in non–fast food restaurants (23). This study and a prior study using the same instrument in an urban environment (17) both found more meals that meet healthy criteria at fast-food restaurants than sit-down restaurants. However, it is unknown whether this was an indication that fast-food restaurants had more healthy options or whether it was a result of lack of nutritional information available, which is needed to assess whether meals met nutrition criteria, at nonchain restaurants. Our study and the urban study also found similar patterns related to pricing, signage, and promotions.

Our findings for convenience and grocery stores are similar to those in a prior urban NEMS-S study in terms of average scores and lower availability and quality in convenience stores than in grocery stores (18). Grocery stores in rural areas offer more healthful food selections but are outnumbered by convenience stores, which not only offer less variety of healthful food choices (24,25) but are also in more accessible locations; these factors lead to a higher frequency of visits by the community, which influences their nutrient intake (26).

Information from this comprehensive assessment of the food environment, in conjunction with information from interviews conducted with restaurants and store owners, is being used to design interventions. Because availability of healthy foods varied in restaurants, a restaurant program was developed using the healthy practices measured in the NEMS-R as a foundation to target practices that needed improvement and determining realistic strategies for restaurants of all levels of healthy practices to achieve. Restaurants with the lower scores, especially the independent ones, because of their flexibility regarding changing menus, were the primary target. Restaurants enrolled in the program, signed a contract and agreed to make certain practices available while the program staff offered nutrition consulting, analysis of recipes, and promotion of restaurants with healthier practices. Restaurants will be reassessed with a revised NEMS using updated nutrition guidelines and a few additional risk factors to inform the next phase of the restaurant program and assess changes. As the HONU project focuses on interventions to improve cardiovascular health, there are some additional measures (eg, sodium, saturated fat, trans fat) that may be important to add to the NEMS tools to further measure risks in the food environment. 

### Limitations

A limitation when interpreting NEMS-R data is related to the nutrition information available to assess whether meals met criteria. Restaurants with nutrition information easily reached higher total scores regardless of nutritional quality of foods served. Generally, only chain restaurants had nutrition information available due to response to changing regulations, and in our rural community, there were many family-owned restaurants. A limitation of the NEMS-S is that it does not assess the deli-prepared salads and meals where consumers shop as an alternative to cooking or eating out. In addition, it does not assess some strategies (eg, product placement) that stores can use to promote and encourage the purchase of healthful food items.

The food environment of this study may not be representative of other rural food environments and may not be translatable to more metropolitan areas. Some components of the food environment (eg, vending machines, worksite cafeterias, farmers markets) were not included. The study also excluded places that were not regularly frequented by the adult population, such as school concessions. The small number of restaurants and stores limited statistical tests, but comparisons provided results for descriptive purposes and to identify potential opportunities for interventions. Chances of interrater differences were minimized by proper training, using the same rater to conduct most of the assessments, and having 2 raters assess a few of the same restaurants for comparison. Finally, results of the surveys represent one point in time and cannot account for previous or future alterations on restaurant menus, seasonal variations in menu or store items, or the emergence of new and changing store formats (27).

### Conclusion

Availability, price, and promotion of healthful food choices in the food environment are important in developing strategies that support people and communities in the prevention of obesity and cardiovascular disease (28). In this study, assessment of the primary nutrition environments (restaurants, grocery stores, convenience stores) helped provide strategic focus for future interventions, particularly in restaurants, being developed as part of a community-based prevention program. Further research and evaluation after the various interventions are implemented will determine if these sets of practices improved the rural nutrition environment and how these practices are linked to changes in consumer food behaviors.
